# A novel augmented reality system for displaying inferior alveolar nerve bundles in maxillofacial surgery

**DOI:** 10.1038/srep42365

**Published:** 2017-02-15

**Authors:** Ming Zhu, Fei Liu, Gang Chai, Jun J. Pan, Taoran Jiang, Li Lin, Yu Xin, Yan Zhang, Qingfeng Li

**Affiliations:** 1 Shanghai Ninth People’s Hospital affiliated to Shanghai Jiao Tong University, Shanghai, China.; 2 State Key Laboratory of Virtual Reality Technology and Systems Beihang University, Beijing, China

## Abstract

Augmented reality systems can combine virtual images with a real environment to ensure accurate surgery with lower risk. This study aimed to develop a novel registration and tracking technique to establish a navigation system based on augmented reality for maxillofacial surgery. Specifically, a virtual image is reconstructed from CT data using 3D software. The real environment is tracked by the augmented reality (AR) software. The novel registration strategy that we created uses an occlusal splint compounded with a fiducial marker (OSM) to establish a relationship between the virtual image and the real object. After the fiducial marker is recognized, the virtual image is superimposed onto the real environment, forming the “integrated image” on semi-transparent glass. Via the registration process, the integral image, which combines the virtual image with the real scene, is successfully presented on the semi-transparent helmet. The position error of this navigation system is 0.96 ± 0.51 mm. This augmented reality system was applied in the clinic and good surgical outcomes were obtained. The augmented reality system that we established for maxillofacial surgery has the advantages of easy manipulation and high accuracy, which can improve surgical outcomes. Thus, this system exhibits significant potential in clinical applications.

Facial bone contouring surgery is one of the most commonly performed aesthetic procedures in Asia. Since Legg’s first report on masseteric hypertrophy^1^, the correction of the prominent mandibular angle via surgery has become a common procedure. Later, Adams and Converse proposed the use of masseter resection for mandibular angle osteotomy^2^, and many related techniques for these procedures have since been presented. However, the complexity of the anatomical structures often impairs the direct visualization of the surgical field in facial bone contouring surgeries. Therefore, correctly understanding the relationship between soft tissue and bone, such as the osteotomy line or the inferior alveolar nerve bundles (IANs), is important in facial skeletal surgery. Improving the visualization of the 3D relationships between these internal structures would substantially improve surgical results.

Innovative imaging technologies in the form of augmented reality (AR) techniques, which can provide surgeons with “integral images,” have been widely adopted in many medical fields, such as orthopedics and ophthalmology^3^,^4^,^5^. Integral images consist of computer-generated information and user’s sensory perceptions to help surgeons visualize sites that cannot be observed by the naked eye. AR provides navigational information from pre-operative data and superimposes it on the surgical site to enhance the perception of the physical environment. Okamota^3^ developed an AR-based navigation system for abdominal surgical procedures that exhibited mean registration errors of approximately 5 mm. Scuderi GR^5^ overlaid virtual images onto real images using an AR technique to obtain a 3D view for total knee arthroplasty and verified that the navigation system could improve surgical accuracy and clinical outcomes. Thus, the AR technique has been considered a useful tool for intra-operational navigation.

However, AR systems have not been applied in maxillofacial surgery. Thus, we studied the fundamental techniques necessary to establish an AR-based navigation system and found that proper registration and tracking techniques are essential for this application.

In this study, we developed a novel registration and tracking technique based on the occlusal relationship to establish an AR system for maxillofacial surgery. Furthermore, using mandibular surgery as an example, we superimposed osteotomy lines and IANs onto the actual surgical field during surgery.

## Materials and Methods

### Subjects

After approval from the institutional review board, the medical records of 20 patients (7 females and 13 males, with a mean age of 34.7 ± 5.04 years) who were surgically treated for mandibular disease between March 2014 and October 2015 were studied. Informed consent was obtained from these patients. All experimental protocols were approved by the Independent Ethics Committee of Shanghai Ninth People’s Hospital affiliated with the Shanghai Jiao Tong University, School of Medicine, and all methods were performed in accordance with the relevant guidelines and regulations.

### System structure

**Figure 1:**
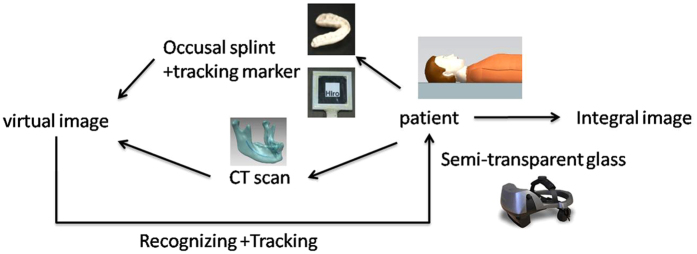
Workflow of our AR system.

The workflow of our system includes several essential parts needed to prepare an “integrated image”: virtual image acquisition, real model manufacturing, and registration. These parts are illustrated in Fig. 1.

### Virtual image acquisition

Virtual image refers to the added navigational information displayed during the surgery. This research was performed to display virtual 3D image of IANs. Several steps were performed to attach the tracking markers to the IAN images.

**Figure 2:**
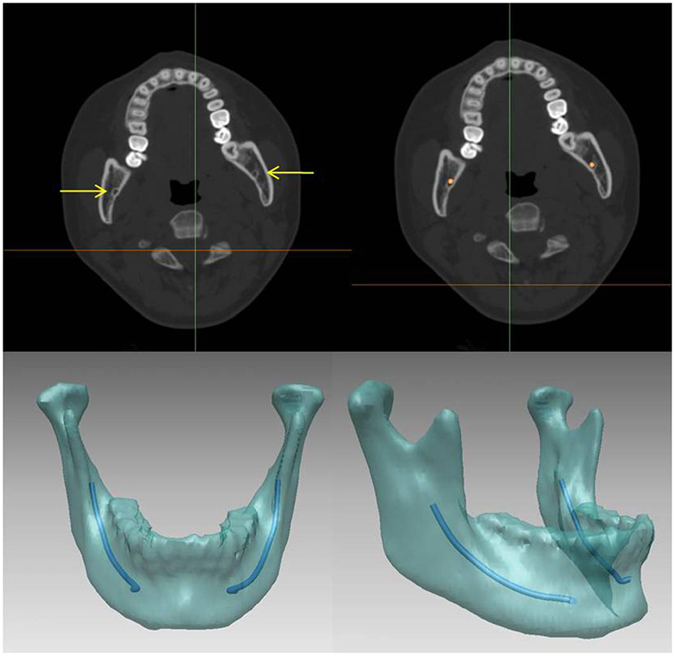
Construction of IAN model: Based on different gray levels, IAN bundles can be visualized independently from bony tissue after segmentation. The figure also shows a reconstructed 3D image of the mandible and IANs by volume rendering.

In the first step, the skull of the patient was scanned using 3D CT (GE LightSpeed 16, Milwaukee, WI). The DICOM image data were imported into the Mimics CAD/CAM software (Materialise, Leuven, Belgium) to generate a 3D model of the skull and surrounding soft tissue. Because the gray levels of IANs and bone differ, they can be visualized independently from bony tissue after segmentation. Accurate 3D images of the structural components, mandible and IANs were then obtained (Fig. 2).

**Figure 3:**
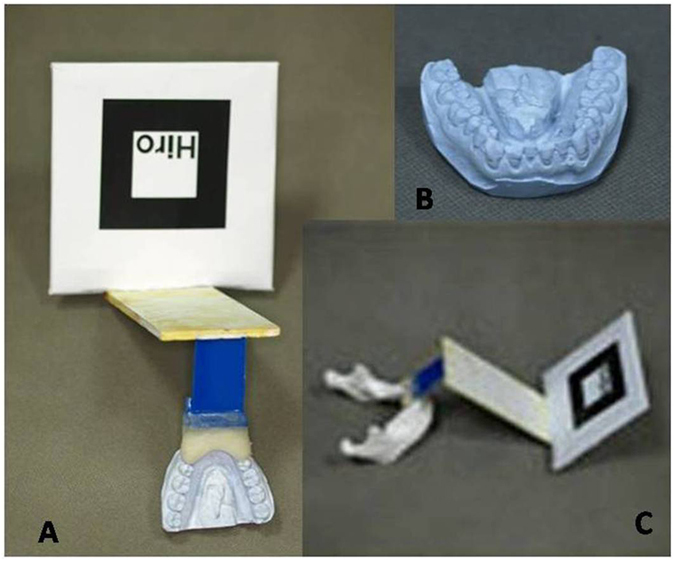
Fabricated dental cast, occlusal splint and cement with the tracking marker. (**A**) Dental cast matching the occlusal splint cemented with the tracking marker; (**B**) dental cast; (**C**) mandible model matching the occlusal splint cemented with the tracking marker.

Dentists produced a dental cast for each patient and modeled occlusal splints based on the central incisors and lateral incisors using autopolymerizing acrylic resin. The occlusal splint was then fixed to a tracking marker, which was recognized by the AR software as a fulcrum. Via the occlusal splint, the marker and mandible maintain a constant relationship during the surgery (Fig. 3). The 3D morphological data of dental casts and tracking marker can be transformed into digital data using a laser scanner (Konica Minolta Vivid 910).

### Integration of virtual image

**Figure 4:**
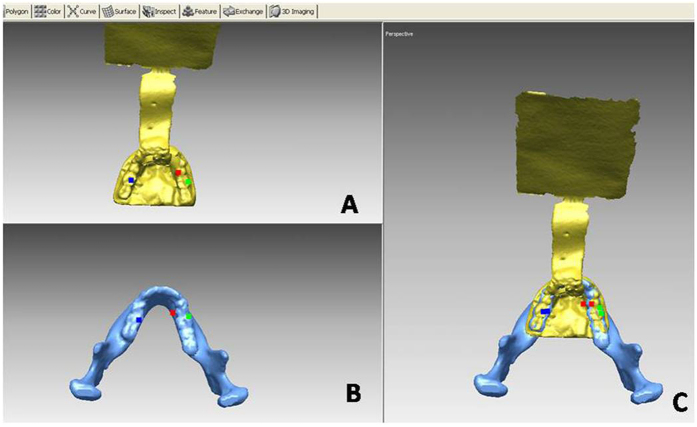
Virtual integration process. The virtual images of the dental cast and mandible were integrated using 3 designated points. (**A**) The scanned 3D model of the occlusal splint and tracking marker; (**B**) the model of the mandible from CT data using volume rendering; (**C**) the well-prepared virtual image for performance in an AR system.

For intra-operational use, the mandible and occlusal splint data obtained from the tracking marker should be integrated into one picture based on the same dental cusps, which are called designated points. The mesiolingual cusp tip of the right mandibular 1st molar, buccal cusp tip of the right mandibular 2nd molar and mesiolingual cusp tip of the left mandibular 1st molar were selected as designated points. These points can be explicitly recognized on the mandible and occlusal splints (Fig. 4). Using this AR process, two parts of a virtual image were integrated into one picture to generate the virtual image for performance. As the virtual image for navigation is obtained using a computer, the patient avoids additional radiation and costly procedures.

### Rapid prototyping of the mandible model

The 3D digital virtual model of the mandible was reconstructed and imported into a Zcorp 510 3D printer (3D Systems, Rock Hill, South Carolina), and a rapid prototyping model of the mandible was then printed. The occlusal splint with the tracking marker was fixed to the 3D printed mandible model before registration.

### 2.5 Registration and tracking

**Figure 5:**
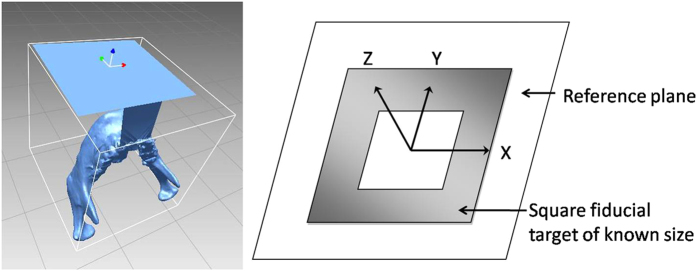
Coordinating the original point of the virtual image to the center of the tracking marker.

Registration and tracking are essential components of an AR system, and these processes require the most computation time during operation of the system. Specifically, these processes combine a virtual image with the real object in a proper position by perfectly superimposing the two. Here, we used the software AR Toolkit to recognize the tracking marker. This software then projected the virtual image into the real environment according to the center of the marker (Fig. 5).

The registration strategy of our system consisted of a noninvasive fiducial marker connected to an occlusal splint. Based on the unique occlusal position, the marker cemented with the occlusal splint maintained a stable position on the patient’s head, which allowed the virtual image and real environment to be bridged both pre-operatively and intra-operatively. The detailed tracking algorithm is described as follows:

1. Set the threshold range value of the tracking marker;
2. Compute the intensity difference between the current frame and the background environment;
3. Evaluate the range support by the difference mask to reduce the noise and shadow;
4. Locate the coordinates (x, y) of the center of the markers using an a priori location estimate provided by the Kalman filter;
5. Use depth data to determine the z-coordinate of the center of the marker;
6. Update the location detected by the Kalman filter and the background model using a different mask;

Before the projection, the original point of the virtual 3D image and real object should be coordinated using Autodesk 3ds Max (version 9) to complete the overlay process. As the tracking marker was firmly fixed to the mandible by an occlusal splint, the virtual IAN image can be continuously tracked based on the mandible.

### Accuracy assessment of the AR system

**Figure 6:**
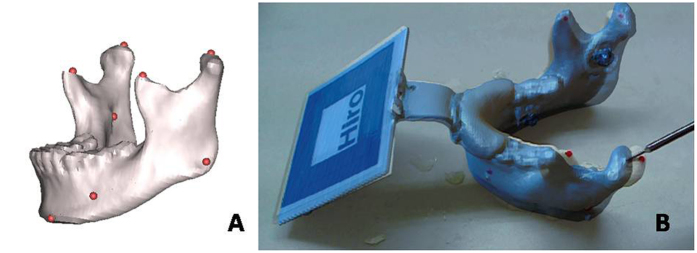
Eleven points marked in red on both 3D virtual mandible and the printed real models. The measuring points marked in red were added to the virtual image (**A**) and are presented on the printed model by AR (**B**).

The precision of this system was studied to ensure its validity for application in maxillofacial surgery. Eleven measurement points (Table 1) were marked on both the 3D reconstructed virtual mandible and 3D printed models (Fig. 6). The virtual and real coordinates of each point were then obtained in the AR environment; here, the deviation in the 3D measuring point coordinates between the 3D printed model and the virtual model in the AR navigation indicates the accuracy of this system. All tests were performed by the same observer, and 3 repeated tests were performed for each case.

### Application in a clinical case

After approval from the institutional review board, 20 patients with craniofacial deformities who were surgically treated at our plastic and reconstructive surgery department between March 2014 and October 2015 were studied.

These patients had been diagnosed with various conditions, including mandibulofacial dysostosis, mandible retrognathism, and mandible hypertrophy, all of which require IAN protection during surgery. Patients whose IANs had been injured before surgery were excluded from this study. We studied the feasibility and accuracy of this AR system for the treatment of these patients. All photographic releases were approved by the patients.

## Results

The AR system was applied to display the IANs of 20 patients (Table 2), and the occlusal splint of each patient was constructed by the same professional dentist. Overall, 20 plastic models were printed by Zcorp based on the patients’ CT data.

**Figure 7:**
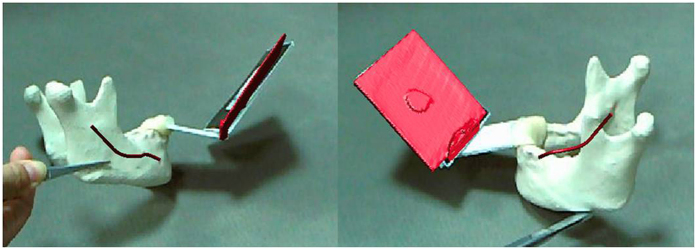
“Integrated image” combining virtual IAN model with printed mandible model.

The experimental results demonstrated that the “integral image” of the virtual IANs and real models could be superimposed in the correct position (Fig. 7). When the mandible moves, the tracking marker and IANs move simultaneously. Using the AR technique, stereopsis is possible from every view position, and the stereoscopic images are available in multiple views.

Once the AR software was running, surgeons required only 1–5 minutes to examine the result of registration before surgery. With the help of the projected virtual IANs, the surgeon was able to precisely operate and avoid damage to important structures.

### Accuracy of this AR system

The accuracy of this system is indicated by the positional error (in mm), which is obtained by measuring the deviation in the 3D coordinates of each measuring point on the 3D printed model and virtual model generated by AR navigation. The positional error ranged from 0.52–2.00 mm (average 0.96 mm; standard deviation 0.51 mm) (Table 3). As these error values are negligible, this AR system is considered highly accurate.

### Feasibility of system application in the clinic

All patient data are summarized in Table 4.

#### Case 1

A 27-year-old male patient visited the plastic and reconstructive surgery department of our hospital complaining of a deviated mandible that was larger on the right side. The patient reported that this condition affected his social life. The clinical examination revealed significant facial asymmetry, with a deviation of the mandible to the left side of the face. Based on a clinical assessment and panoramic radiographs, the patient was diagnosed with a typical hemimandibular hyperplasia, which presented solely with a marked increase in the right mandibular body weight without condyle hyperplasia.

An osteotomy was planned to correct his facial asymmetry, requiring the locations of IANs to be accurately determined before the surgery to reduce the surgical complications. After the reconstruction of the skull and IANs of the patient, a presurgical design was generated, and the osteotomy lines were presented by the AR system during the surgery to guide the actual surgery.

**Figure 8:**
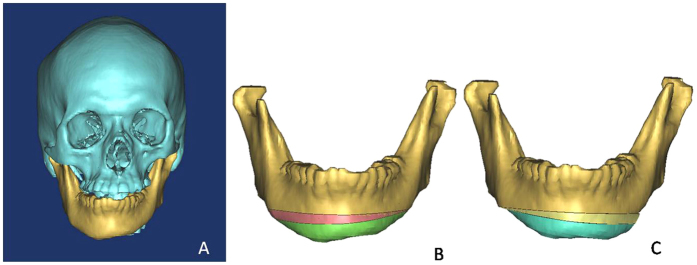
Surgical plan with the 3D model of bony tissue. (**A**) 3D model of the patient’s skull; (**B**) two osteotomy lines were drawn to split the deviated mandible into three parts; (**C**) to ensure a symmetric mandible margin, the intermediate bone section (light yellow) was rotated 180° and fixed to the upper (golden) and lower parts (blue).

The surgical plan was designed pre-operatively for the patient to correct the facial deformity according to the standard procedure and measurement data (Fig. 8). On the virtual planning platform, we drew two osteotomy lines to split the mandible into three parts and then merged these parts after the intermediate bone section was rotated 180° to achieve a symmetric mandibular margin.

**Figure 9:**
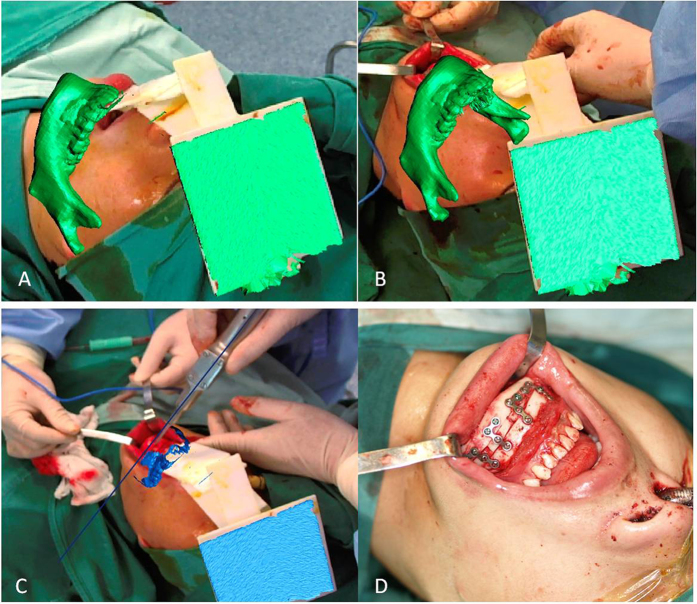
AR technique applied during surgery. (**A**) The integrated image combined a virtual mandible and the patient to provide a “see-through” experience for surgeons. (**B**) After the mandible was exposed, the virtual image of the mandible was completely overlaid with the real bone in 3D. (**C**) The surgery was conducted according to the osteotomy lines projected by the AR system. (**D**) Internal rigid fixation with L-shaped plates and screws was used to fix the three parts of the mandible.

To precisely guide the surgery, we used our AR system during the surgery to present the osteotomy lines. Figure 9 shows the navigation image used to correct the mandibular deformity. Using the AR system, the virtual image of the cutting plane reconstructed by CT data was successfully superimposed on the patient and fully overlaid with the real bone during the surgery. Osteotomy was performed under the guidance of an AR system, and internal rigid fixation with L-shaped plates and screws was used to fix the three parts of the mandible.

**Figure 10:**
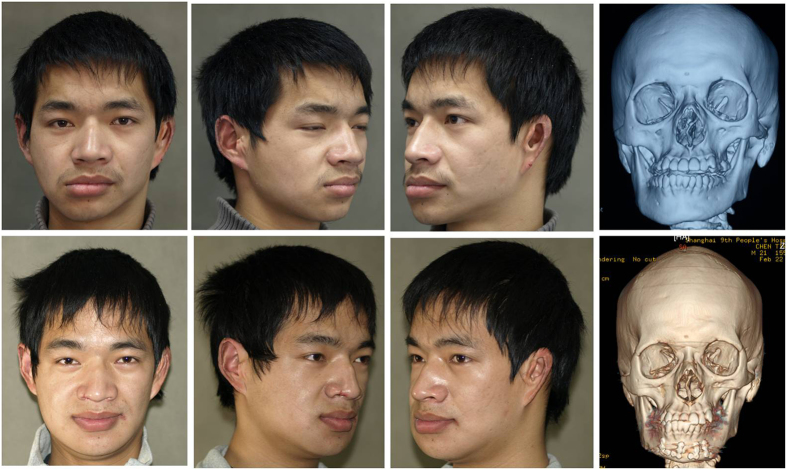
Photographs and computed tomographic (CT) scans before surgery (above) and three months after surgery (below) revealed a significant improvement in the facial symmetry.

Three months after surgery, CT scans showed that the previously observed asymmetry had been corrected. The patient was satisfied with his appearance (Fig. 10).

#### Case 2

A 30-year-old female patient visited our plastic surgery department complaining of mandibular angle hypertrophy, which is considered unattractive in China. The patient reported that both sides of her mandibular angle were unusually large, and she had no history of trauma or inflammation in the jaw or in the temporomandibular joint.

**Figure 11:**
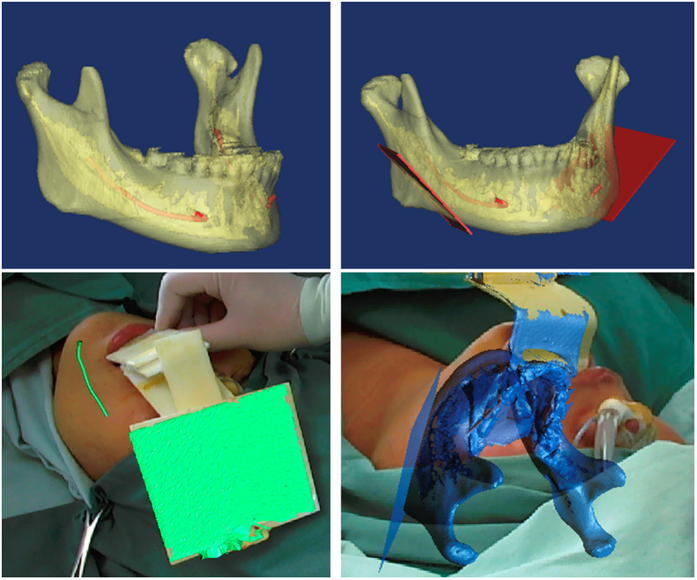
The IANs were reconstructed using a 3D technique before surgery (above left), and osteotomy lines were drawn far from the IANs (above right). Using the AR system, the IAN image was displayed on the surgical site (below left), and osteotomy lines could be clearly observed during the surgery (below right).

After consulting with the patient, a mandibular angle osteotomy was planned. Computer tomography pre-operatively revealed the location of IANs, and the osteotomy lines were drawn far from the IANs. Moreover, our AR system explicitly showed the IANs and osteotomy lines during surgery to protect the IANs from injury (Fig. 11).

**Figure 12:**
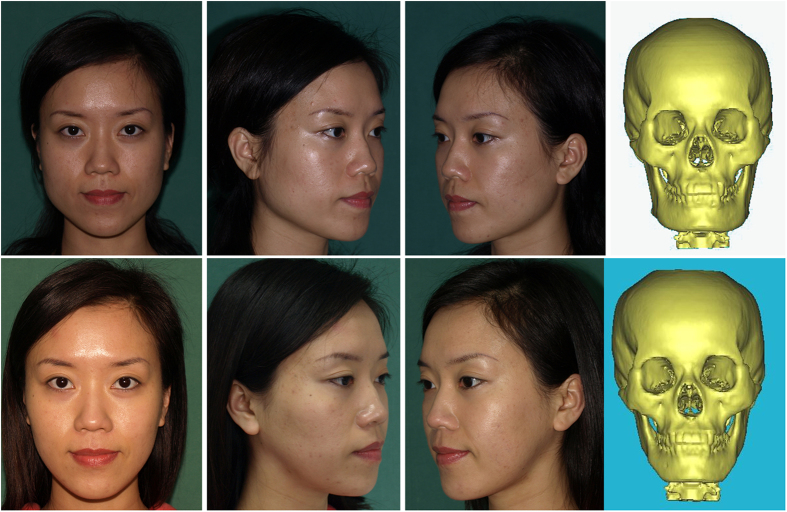
Photographs and computed tomography (CT) scans before surgery (above) and four months after surgery (below) revealed a satisfactory outcome of the treatment for mandibular angle hypertrophy.

Four months after surgery, patient follow-up was conducted via CT scans and photographs. The pictures showed the correction of mandibular angle hypertrophy, and the patient was satisfied with the outcome (Fig. 12).

These cases demonstrate that the AR technique provides intuitive visualization for maxillofacial surgery. The integrated image, which combined the virtual information and the real environment, is helpful during all phases of surgery.

## Discussion

Intraoperative navigation systems can help surgeons to accurately perform surgeries and improve surgical results by displaying a virtual image designed before surgery. The benefits of image-guided surgery have been reported in several studies, and AR is currently being investigated for use in various surgical fields, including orthopedics^6^, spine surgery^7^, laparoscopic surgery^8^, neurosurgery^9^, and biopsy procedures^10^,^11^,^12^. Specifically, several authors describe the use of AR systems in craniomaxillofacial surgery to reconstruct post-traumatic defects^13^, analyze temporomandibular joint motion^14^, and resect tumors^15^. Visualization technologies improve the orientation during and safety of surgery. Although conventional image-guided surgery provides a 3D view of the patient’s anatomy, precisely correlating the displayed image with the surgical field is difficult and necessitates continuous comparisons between the surgical field and the displayed image that involve many hand-to-eye modifications^16^. Due to the complex 3D geometry and the need for precise facial symmetry, an appropriate image-guided system is required to display IANs during maxillofacial surgery.

AR fundamentally differs from conventional image-guided techniques because the AR system transparently displays the 3D image directly on the surgical site; in addition, the tracking marker enables the real-time tracking of mandible movements, which allows the 3D images to be updated in real time during surgery. Thus, AR systems provide navigation support by directly projecting segmented structures from pre-operative images onto the surgical field.

As the name implies, AR is a mixed medium that overlays synthetic information atop real images to assist the user. Conventional image-guide techniques are completely immersive, meaning the user operates completely within the virtual environment and lacks direct interactions with the real environment. Conversely, in an AR system, the user continues to operate within the real-world environment^17^. In other words, conventional image-guide techniques aim to replace the real world, whereas AR supplements it^18^. Because AR systems project information directly onto the surgical site for navigation, attention does not need to be alternated between the surgical site and the monitor.

The key challenge for all surgical navigation is the registration of the virtual image to the real model^19^, and AR systems face the same problem. Precise registration is crucial because it has direct repercussions on the precision of all subsequent navigation tasks. Thus, finding an appropriate way to register the image for maxillofacial surgery is important.

The registration process allows the computer to integrate the spatial information of the actual patient (Cartesian coordinates x, y, z) with the virtual patient (Cartesian coordinates x’, y’, z’) to precisely localize the patient in space.

All AR systems require a fiducial marker to transmit the virtual image to a real image. Specifically, marker-based registration requires markers that are apparent in the pre-operative images and are easily detectable in the patient during intraoperative registration^20^. The registration methods can be separated into 2 types: invasive and noninvasive. Invasive fiducial markers, such as titanium screws, are rigidly fixed to the skull before the pre-operative CT scan and remain in place until the procedure is complete. Noninvasive fiducial markers can be subdivided into three types: adhesive markers, dental appliances, and anatomic landmarks (for example, bone and the skin surface^21^). In this study, we selected a dental appliance, such as a dental cast, as a fiducial marker during AR based on the features of maxillofacial surgery. This method proved to be sufficiently accurate for guidance in this application.

In our technique, a dental reference splint was fixed to a real rapid prototyping model, and 3D dental casts provided registration between imaging data and the patient. The splint was mounted before image acquisition and then removed and replaced before each registration. A similar process has been reported by Nijmeh AD^22^ *et al*., and the measured target registration errors illustrate an acceptable accuracy of approximately 1 mm for clinical deployment in the peri-orbital mid-face region. Specifically, 3D data describing the dental cast, mandible and IAN bundle region of the patient are integrated with specialized software. For maxillofacial surgery, the head is fixed in place by conventional methods. During oral surgery, patients sometimes move their head during surgery or open/close their mouth, which moves the jaw. As the dental reference splint is mounted stably on the lower tooth, the marker moves automatically with the jaw under such circumstances. Thus, our surgical navigation accommodates movement.

The positional error of the AR system presented in this paper was 0.96 ± 0.51 mm, which can be considered negligible. Thus, the technique is highly accurate. This system has already been applied in maxillofacial surgeries at our hospital’s clinic.

The advantage of the presented system relative to conventional navigation systems is the direct and improved visualization of regions of interest in the patient’s head. Furthermore, this system is inexpensive and easy to reproduce. Thus, it may be beneficial for surgeons or hospitals that cannot afford an expensive navigation system. Moreover, we used a laser scanner to obtain 3D information from the dental cast with a real model, which avoided the need for a second CT scan with the splint in place.

However, this system also features several limitations to be considered:

1. Specialized skills are required to fabricate the splint;
2. Fabrication of the splint requires time, which delays the time to surgery;
3. Loosening or incorrect placement of the occlusal splint, either during imaging or registration, can result in unexpected errors^23^.
4. Occlusal splints cannot provide satisfactory results when oral stabilization is challenging, such as for edentulous patients^24^. Nevertheless, we consider dental splints useful for precise registration. Overall, the AR system improved surgical precision and optimized results.

## Conclusion

We presented the application of a 3D-imaging AR system in the field of maxillofacial surgery. An experimental accuracy analysis and case studies showed that IANs could be visualized directly with correct stereo-positioning. The novel AR system that we established could “see through” the bone structure and improve the results of maxillofacial surgery in the clinic.

## Additional Information

**How to cite this article**: Zhu, M. *et al*. A novel augmented reality system for displaying inferior alveolar nerve bundles in maxillofacial surgery. *Sci. Rep.* **7**, 42365; doi: 10.1038/srep42365 (2017).

**Publisher's note:** Springer Nature remains neutral with regard to jurisdictional claims in published maps and institutional affiliations.

## Figures and Tables

**Table 1:** Definition of all mandibular landmarks.

Abbreviation	Definition
MP-L	Medial pole of condyle of left side
MP-R	Medial pole of condyle of right side
CP-L	Coronoid process of left side
CP-R	Coronoid process of right side
Go-L	Gonion of left side
Go-R	Gonion of right side
MeF-L	Mental foramen of left side
MeF-R	Mental foramen of right side
MaF-L	Mandibular foramen of left side
MaF-R	Mandibular foramen of right side
C	Chin

**Table 2:** Patients’ demographics.

No. of patients	20
Mean age at surgery, y	34.7 ± 5.04 (range 26–38)
Sex of patients	13 M/7 F
Duration of hospital stay, days	9.3 ± 11.4

**Table 3:** Average positional error at these measuring points (mm).

Measuring point	Average positional error
MP-L	1.57 ± 0.14
CP-L	1.04 ± 0.31
Go-L	0.85 ± 0.15
MeF-L	0.56 ± 0.13
MaF-L	0.56 ± 0.07
C	0.52 ± 0.22
MaF-R	0.55 ± 0.14
MeF-R	0.66 ± 0.25
Go-R	0.84 ± 0.34
CP-R	1.33 ± 0.11
MP-R	2.00 ± 0.15

**Table 4:** Summary of patient data.

Patient number	Age (y)	Gender	Disease	Surgery received	Complications
1	35	Female	Mandibular angle hypertrophy	Mandibular angle osteotomy	None
2	26	Male	Hemifacial microsomia	Mandibular distraction osteogenesis	None
3	30	Female	Mandibular angle hypertrophy	Mandibular angle osteotomy	None
4	35	Male	Mandible asymmetry	Mandible plasty	None
5	37	Male	Mandible asymmetry	Mandible plasty	None
6	29	Male	Hemifacial microsomia	Mandibular distraction osteogenesis	None
7	34	Female	Mandibular angle hypertrophy	Mandibular angle osteotomy	None
8	27	Male	Laterognathism	Genioplasty	None
9	33	Male	Mandibular angle hypertrophy	Mandibular angle osteotomy	None
10	37	Female	Mandibular angle hypertrophy	Mandibular angle osteotomy	None
11	35	Male	Hemifacial microsomia	Mandibular distraction osteogenesis	None
12	38	Female	Mandibular angle hypertrophy	Mandibular angle osteotomy	None
13	32	Female	Mandibular angle hypertrophy	Mandibular angle osteotomy	None
14	36	Male	Hemifacial microsomia	Mandibular distraction osteogenesis	None
15	34	Male	Mandible asymmetry	Mandible plasty	None
16	38	Male	Mandibular angle hypertrophy	Mandibular angle osteotomy	None
17	37	Male	Mandible asymmetry	Mandible plasty	None
18	36	Male	Laterognathism	Genioplasty	None
19	38	Female	Mandibular angle hypertrophy	Mandibular angle osteotomy	Hematoma
20	36	Male	Laterognathism	Genioplasty	None
